# Effects of Low Frequency Stimulation on Spontaneous Inhibitory and Excitatory Post-Synaptic Currents in Hippocampal CA1 Pyramidal Cells of Kindled Rats

**DOI:** 10.22074/cellj.2016.4721

**Published:** 2016-09-26

**Authors:** Samireh Ghafouri, Yaghoub Fathollahi, Saeed Semnanian, Amir Shojaei, Javad Mirnajafi-Zadeh

**Affiliations:** Department of Physiology, Faculty of Medical Sciences, Tarbiat Modares University, Tehran, Iran

**Keywords:** Seizure, Post Synaptic Potential, Low-Frequency Stimulation, Kindling

## Abstract

**Objective:**

Low-frequency stimulation (LFS) exerts suppressive effects in kindled animals. It is believed that overstimulated glutamatergic and decreased GABAergic transmission have long been associated with seizure activity. In this study, we investigated the effect of electrical LFS on different parameters of spontaneous excitatory and inhibitory post-synaptic currents (sEPSCs and sIPSCs) in hippocampal CA1 pyramidal cells in kindled animals.

**Materials and Methods:**

In this experimental study, rats were kindled by electrical stimulation of the hippocampal CA1 area in a semi-rapid manner (12 stimulations/day). The animals were considered fully kindled when they showed stage 5 seizures on three consecutive days. One group of animals received LFS 4 times at 30 seconds, 6 hours, 18 and 24 hours following the last kindling stimulation. Each LFS consisted of 4 packages at 5 minutes intervals. Each package of LFS consisted of 200 pulses at 1 Hz and each monophasic square wave pulse duration was 0.1 millisecond. At 2-3 hours post-LFS, acute hippocampal slices were prepared and a whole cell patch clamp recording was performed in all animals to measure the different parameters of sEPSCs and sIPSCs.

**Results:**

In kindled animals, the inter-event interval (as an index of occurrence) of sEPSCs decreased, whereas sIPSC increased. In addition, the decay time constant of sIPSCs as an index of the duration of its activity decreased compared to the control group. There was no significant difference in other parameters between the kindled and control groups. Application of LFS in kindled animals prevented the observed changes. There was no significant difference between the measured parameters in kindled+LFS and control groups.

**Conclusion:**

LFS application may prevent seizure-induced increase in the occurrence of sEPSCs and seizure-induced decrease in occurrence and activity duration of sIPSCs.

## Introduction

Epilepsy is a common neurological disease characterized by repeated spontaneous seizures (1). Kindling is the most commonly used model of temporal lobe epilepsy which can be generated by repetitive, low-intensity electrical stimulation of limbic areas including the hippocampus (2,3). Excessive activation of the excitatory pathways in the brain, including glutamate transmission and an imbalance between neuronal excitation and inhibition, is believed to be the contributing mechanism involved in epileptogenesis. It is well known that the increase in activation of glutamate receptors has a role in initiation and spread of epileptic discharges during the kindling procedure (4). Moreover, N-methyl-D-aspartate (NMDA) receptor antagonists are effective anticonvulsants (5). This overstimulated glutamatergic transmission, mediated primarily through the NMDA and other glutamatergic ionotropic receptors, significantly contributes to the development of the kindling phenomenon (4). 

It is well established that activation of postsynaptic GABA_A_ receptors mediates fast inhibitory post-synaptic currents (IPSCs) caused by Clconductance. A decrease in GABA_A_ neurotransmission has long been associated with seizure activity so that GABA_A_ receptor antagonists induce seizures (6) and drugs that enhance GABA_A_ receptor-mediated neurotransmission have anticonvulsant effects (7,8). Acute seizures suppress GABA_A_ receptor function (9,10) and animals with epilepsy are associated with changes in GABA release, immunoreactivity, and binding sites (11,14). 

Despite the high prevalence of epilepsy, there is still no absolute treatment for epileptic patients. Anticonvulsant medication, as the most common therapeutic method, can only suppress seizures rather than correct the underlying brain abnormalities that cause epilepsy (15). Therefore, it is necessary to find new ways for reducing the seizure-induced impairments in the brain. 

Electrical low-frequency stimulation (LFS) is effective against kindled seizures (16,17). Despite numerous investigations that show the potential therapeutic effect of LFS on epileptic seizures, the cellular consequences that underlie these effects are still unknown. It has been shown that the inhibitory effect of LFS on kindling development is accompanied with a decrease in seizure-induced hyperexcitability of hippocampal CA1 neurons (18). However the effect of LFS on excitatory (glutamatergic) and inhibitory (GABAergic) synaptic transmission has not been determined. Therefore, in the present study we attempted to investigate whether application of LFS could prevent the kindling-induced changes in occurrence of spontaneous excitatory post-synaptic currents (sEPSC) and sIPSC in CA1 hippocampal pyramidal cells. 

## Materials and Methods

In this experimental study, male Wistar rats (5-6 weeks at the time of surgery) were housed individually in cages with an ambient temperature of 22-25˚C and a 12-hour light/12-hour dark cycle (lights on from 6:00 am-6 pm). Animals received water and food ad libitum. All experimental and animal care procedures were performed according to international guidelines on the use of laboratory animals and approved by Tarbiat Modares University Ethical Committee for Animal Research which is in line with the "NIH Guide for the Care and Use of Laboratory Animals". Efforts were made to minimize both the number of animals used and their suffering. 

### Animal surgery

The rats were deeply anesthetized by a mixture of ketamine and xylazine (Sigma, England, 100/10 mg/ kg, intraperitoneal), fixed in a stereotaxic frame with their skulls exposed. A bipolar stimulating electrode and a monopolar recording electrode were twisted together and implanted in the hippocampal CA1 region of the right hemisphere at 2.4 mm posterior and 1.8 mm lateral to the bregma and 2.8 mm below the skull of each rat (19). In the kindling model of epilepsy the occurrence of after discharges (ADs) at the AD threshold is necessary for seizure progression (2). Therefore, it is essential to record ADs to ensure that the kindling procedure is developed correctly. The teflon-coated, stainless steel electrodes (A-M Systems, Inc., WA, USA, 127 μm in diameter) were insulated except at their tips. A monopolar electrode connected to a stainless steel screw was also positioned in the skull above the occipital cortex as a reference and/ or ground electrode. The electrode location was histologically confirmed in the animals at the end of the experiments. Rats were deeply anesthetized and perfused with 4% paraformaldehyde in 0.1 M phosphate buffer (Sigma, England, pH=7.4). Then, their brains were removed and sectioned to verify electrode placement. 

### Kindling procedure

As described previously (20), the AD threshold was determined following a 7-day post-surgical recovery period. For this purpose, a monophasic square wave with pulse duration of 1 millisecond and the frequency of 50 Hz was applied for 3 seconds. Briefly, the stimulating currents were initially delivered at 30 µA and then its intensity was increased in increments of 10 µA at 10 minute intervals until ADs of at least 8 seconds were recorded. The ADs were considered as spikes whose amplitudes were more than twice the baseline activity and their frequencies were at least 1 Hz. Rats were electrically stimulated at the AD threshold 12 times per day with at an interval of 10 minutes. Epileptiform ADs were continuously recorded from the hippocampal CA1 area following kindling stimulations using a PC-based data acquisition system (D3107, ScienceBeam Co., Iran). 

The behavioral seizure severity was rated according to Racine’s scale (21): stage 0 (no convulsions), stage 1 (facial automatism), stage 2 (head nodding), stage 3 (unilateral forelimb clonus), stage 4 (bilateral forelimb clonus), and stage 5 (rearing, falling and generalized convulsions). The animals were considered fully kindled when they exhibited stage 5 seizure activities over three consecutive days. 

### Low-frequency stimulation application

LFS was applied 4 times at 30 seconds, 6 hours, 18 and 24 hours following the last kindling stimulation. Each LFS consisted of 4 packages at 5 minutes intervals. Each package of LFS consisted of 200 pulses at 1 Hz and each monophasic square wave pulse had a duration of 0.1 milliseconds. The intensity of LFS was equal to the AD threshold of each animal. The LFS pattern was achieved according to our preliminary experiments. 

### Slice preparation and solutions

Because the previous studies showed significant changes in the NR2A subunit of NMDA, GLUR2 subunit of AMPA and γ_2_ subunit of GABA_A_ receptors at 24 hours following kindling stimulations (22,25), we performed slice preparation at 24 hours after kindling. Rats were killed by decapitation while anesthetized with ether. Then, the right hemisphere were rapidly removed and submerged in ice-cold cutting solution that contained 2.5 mM KCl, 0.5 mM CaCl_2_ , 2 mM MgCl_2_ , 1 mM NaH_2_PO_4_ , 26.2 mM NaHCO_3_ , 238 mM sucrose, and 11 mM D-glucose and bubbled with 95% O_2_ 5% CO_2_ . The osmolarity was adjusted to 290-300 mOsm. We used a vibratome (1000 Plus Sectioning System, Vibratome, MO, USA) to prepare the transverse slices (400 μm). Subsequently, the right hippocampi were dissected out and transferred to standard artificial cerebro-spinal fluid (ACSF) (continuously bubbled with 95% O_2_ 5% CO_2_) that consisted of 125 mM NaCl, 3 mM KCl, 1.25 mM NaH_2_PO_4_ , 25 mM NaHCO_3_ , 10 mM D-glucose, 2 mM CaCl_2_ , and 1.3 mM MgCl_2_ . The osmolality was in the range of 290-300 mOsm and pH was adjusted to 7.2-7.35 by 1 M NaOH. Slices were then incubated for one hour at 35˚C and stored at room temperature (23-25˚C) until they were individually transferred to a submerged recording chamber. All chemicals were purchased from Sigma, England. 

### Whole-cell patch clamp recording

For whole cell patch clamp recording, we used a fixed-stage upright microscope (Axioskop 2 FS MOT, Carl Zeiss, Germany) in which a Plexiglas recording chamber was mounted. The chamber was continuously perfused with ACSF at 1.52.5 ml/minutes. All recordings were performed at room temperature (23-25˚C). To visualize the pyramidal cells in the hippocampal CA1 area, an IR-CCD camera (IR-1000, MTI, USA) with a ×40 water immersion objective lens was used. The recorded cells were selected according to their relative pyramidal shape with a smooth, low-contrast appearance. Whole cell patch clamp recordings were made under the voltage clamp mode. Recording microelectrodes (1.5 mm outer diameter, borosilicate glass, GC150-11, Harvard Apparatus, UK) were pulled with a horizontal puller (P-97, Sutter Instruments, USA) and filled with intracellular solution that contained 135 mM K-gluconate, 20 mM KCl, 10 mM HEPES, 0.2 mM ethylene glycolbis(β-aminoethyl ether)-N,N,N',N'-tetraacetic acid (EGTA), 7 mM disodium-phosphocreatine, 2 mM MgATP, 0.3 mM NaGTP and 1 mM QX-314 for the sEPSC recording. For the sIPSC recording, the intracellular solution contained 140 mM CsCl, 1 mM CaCl_2_ , 5 mM QX-314, 10 mM HEPES, 2 mM MgCl_2_ , 2 mM Mg-ATP, 2 mM Na-GTP, and 0.5 mM EGTA. The pH was adjusted to 7.2-7.35 and osmolality was in the range of 290-300 mOsm. All chemicals were purchased from Sigma, England. 

Electrode tip resistance in the bath was 5 to 8 MΩ and series resistance ranged from 18 to 30 MΩ. Cells were rejected if the series resistance changed more than 20% during the experiment. Capacitance compensation and bridge balance were performed. Data were low-pass filtered at 3 kHz and acquired at 10 kHz with a Multiclamp 700B amplifier equipped with a Digidata 1440 A/D converter (Molecular Devices, CA, USA). The signal was recorded on a PC for offline analysis using MiniAnalysis software. After establishment of a gigaseal (more than 2 GΩ), the whole-cell configuration was attained simply by applying a brief suction. 

We investigated the effect of the LFS application in kindled animals on sEPSC and sIPSC of CA1 pyramidal neurons by recording sEPSC and sIPSC of CA1 pyramidal cells for 10 minutes in voltage clamp mode (V=-65) at least 10 minutes after achieving the whole cell configuration. The measured parameters of sEPSC and sIPSC were amplitude, rise slope, decay time constant, and inter-event interval. 

### Experimental design

Animals were assigned to 5 groups. In the kindled group, slice preparation was made at 24 hours following the last kindling stimulation. In the kindled+LFS group, the slice preparation was performed at 2-3 hours after the LFS application. In the control+LFS group, animals were manipulated similar to the kindled+LFS group, however they received only LFS (without kindling stimulations). Another group of animals underwent surgery but did not receive any stimulation. They were considered the sham group. In this group, the elapsing time between surgery and electrophysiological experiments was similar to animals of the kindled and/or kindled+LFS groups (about 15 days). In the control group, electrophysiological experiments were performed on intact animals. 

### Statistical analysis

Data were averaged and expressed as mean ± SEM. was performed using GraphPad Prism version 6.01 for Windows (GraphPad Software, Ca, USA). To evaluate the effect of kindling and LFS application on different parameters of sEPSC and sIPSC, we used two-way ANOVA followed by post-hoc Tukey’s test to compare different parameters of sEPSC and sIPSC in different groups. A P value of less than 0.05 was considered to represent a significant difference. 

## Results

There was no significant difference in AD threshold (82.73 ± 10.37 µA in kindled and 71.75 ± 7.73 µA in kindled+LFS group) and after discharge duration (ADD) after the first kindling stimulation (23.85 ± 1.41 seconds in kindled and 22.88 ± 1.6 seconds in kindled+LFS group) among different experimental groups. In addition, the mean number of stimulation days to achieve the fully kindled state was similar in kindled (9.0 ± 0.39 days) and kindled+LFS (8.18 ± 0.36 days) groups. Therefore, the seizure susceptibility did not differ among experimental groups at the beginning of the experiments. LFS alone had no significant effect on the different parameters of sEPSCs and sIPSCs in CA1 pyramidal cells. There was no significant difference in sham and control groups, thus, their data were merged and considered as the control. 

**Fig.1:**
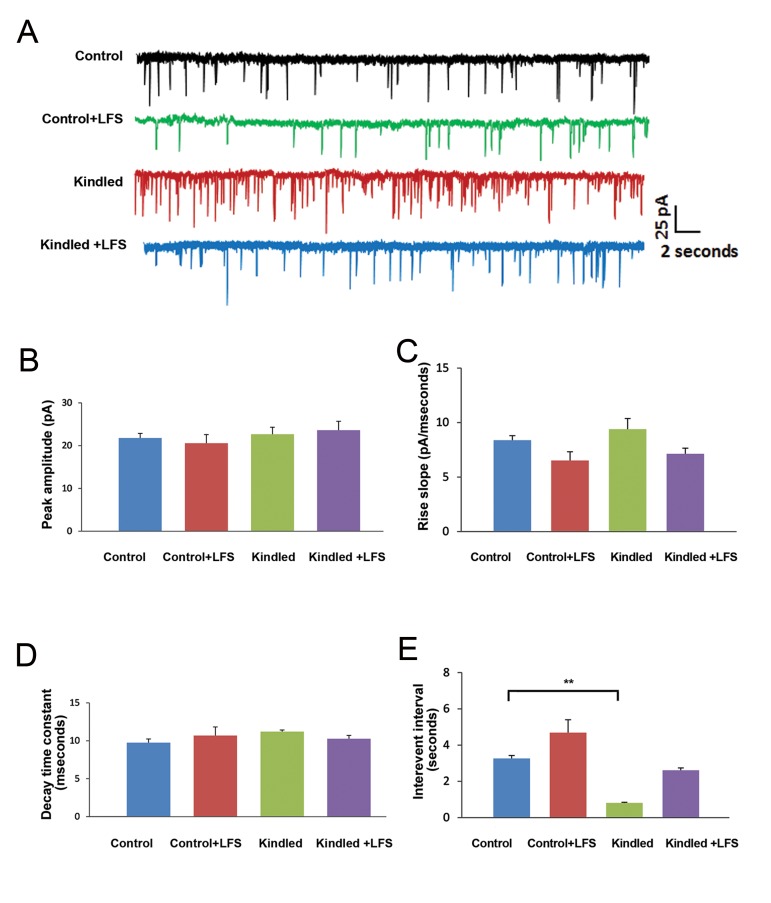
Effect of low-frequency stimulation (LFS) application on kindling-induced changes in spontaneous excitatory post-synaptic currents (sEPSCs) in CA1 hippocampal slices. A. Sample records of sEPSCs in different experimental groups. B-E. Amplitude, rise slope, decay time constant and inter-event interval of sEPSCs in different experimental groups. A significant decrease existed in the interevent interval in the kindled compared to the control group. Application of LFS in kindled+LFS animals prevented the reduction in this parameter. Data are shown as mean ± SEM. **; P<0.01.

In the first experiment we examined the effect of LFS application on sEPSC in pyramidal cells of kindled animals. For the sEPSC recording the voltage was clamped on -65 mV and we added bicuculline (20 µM, Tocris Bioscience, England) as a GABA_A_ receptor antagonist to ACSF. Two-way ANOVA showed no significant difference in peak amplitude, rise slope, and decay time constant of sEPSC among the different experimental groups (Fig.1). However, there was a significant decrease in the inter-event interval of sEPSCs in pyramidal cells in kindled rats (0.8 ± 0.42 seconds) compared to the control group (3.26 ± 1.62 seconds, P<0.01). Application of LFS in kindled animals (kindled+LFS group) prevented this change, so there was no significant difference between this group (2.61 ± 1.52 seconds) and the control group. Application of LFS alone in the control group had no significant effect on this parameter (4.68 ± 1.91 seconds) compared to the control group (Fig.1E). 

**Fig.2:**
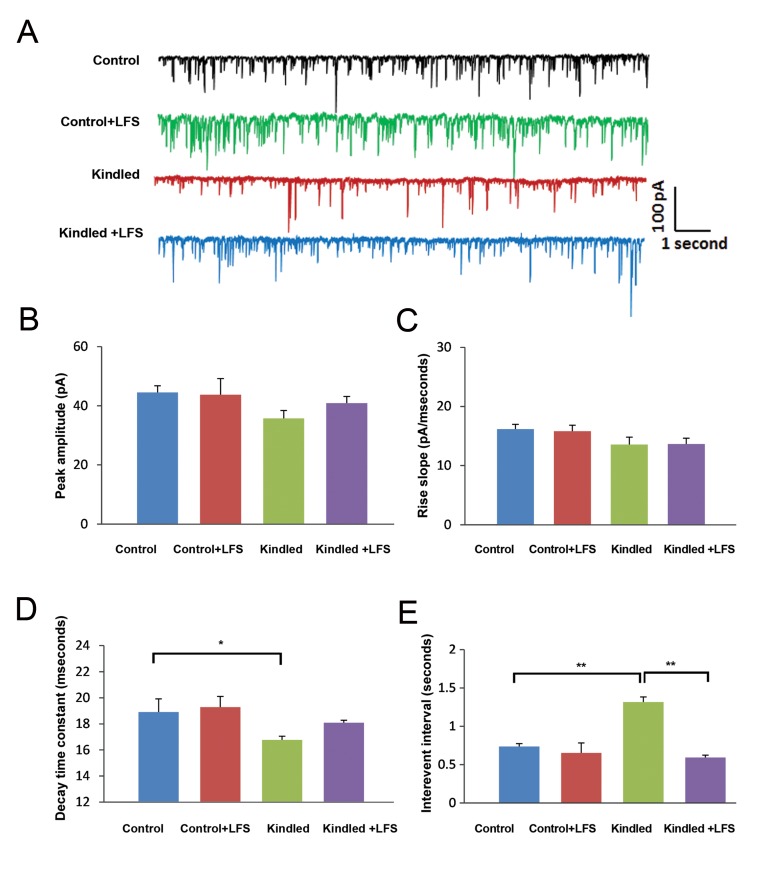
Effect of low-frequency stimulation (LFS) application on kindling-induced changes in spontaneous inhibitory post-synaptic currents (sIPSCs) in CA1 hippocampal slices. A. Sample records of sIPSCs in different experimental groups. B-E. Amplitude, rise slope, decay time constant and inter-event interval of sIPSCs in different experimental groups. The inter-event interval increased whereas the decay time constant decreased in the kindled compared to the control group. Application of LFS in the kindled+LFS group prevented these changes. Data are shown as mean ± SEM. *; P<0.05 and **; P<0.01.

In the second experiment we examined the effect of LFS application on sIPSC in pyramidal cells of kindled animals. For the sIPSC recording the voltage was clamped on -65 mV and we added AP5 (NMDA receptor antagonist, 50 µM, Tocris Bionscience, England) and CNQX (AMPA receptor antagonist, 20 µM, Tocris Bionscience, England) to ACSF. Two-way ANOVA showed no significant difference in peak amplitude and rise slope of sIPSC among the different experimental groups (Fig.2). In contrast to sEPSCs, there was a significant increase in the inter-event interval of sIPSCs in pyramidal cells in kindled rats (1.31 ± 0.30 seconds) compared to the control group (0.73 ± 0.39 seconds, P<0.01). Application of LFS in kindled animals (kindled+LFS group) prevented the increase in this parameter, so that there was no significant difference between this group (0.59 ± 0.33 seconds) and control animals (P<0.01). In addition, the decay time constant of sIPSCs in the kindled group (16.77 ± 0.89 milliseconds) significantly decreased compared to the control group (18.91 ± 1.86 milliseconds, P<0.05). However, there was no significant difference in this parameter between the kindled+LFS (18.08 ± 0.56 milliseconds) and control groups. 

Application of LFS alone in the control group had no significant effect on the inter-event interval (0.65 ± 0.35 seconds) and decay time constant (19.28 ± 2.17 milliseconds) compared to the control group (Fig.2D,E). 

In the present study the excitation/inhibition (E/I) ratio was not measured in a single cell. However, when we calculated this ratio according to the group data of sEPSC and sIPSC parameters, an increased E/I ratio in the kindled compared to control group existed for amplitude (0.63 vs. 0.48) and rise slope (0.70 vs. 0.51). Application of LFS in the kindled+LFS group decreased these ratios toward the control values of 0.57 for amplitude and 0.52 for rise slope.

## Discussion

Our findings indicated that application of LFS in the CA1 region of the dorsal hippocampus could prevent kindled-seizure induced changes in sEPSCs and sIPSCs in pyramidal cells of the hippocampal CA1 area of fully kindled animals. sEPSCs and sIPSCs have been recorded as the indices of neuronal excitability and spontaneous firing. Therefore, in order to record such currents there is no need to stimulate specific neuronal circuits. Epileptic seizures result from many different pathologic processes that disrupt the balance between excitation and inhibition (4). Evaluation the sEPSC and sIPSC parameters is a suitable way to investigate excitability of the nervous system. Anticonvulsant agents are expected to return the activity of excitatory and inhibitory circuits to a normal situation. In this regard, it has been suggested that kindling-like stimulus patterns produce a reduction of GABAergic inhibition in the hippocampus that results from a stimulus-induced postsynaptic activation of NMDA receptors. The NMDA receptor-dependent changes in GABAergic inhibition may be involved in kindling-induced synaptic plasticity (10). 

The results of this study showed a significant decrease in inter-event interval of sEPSCs in pyramidal cells of the CA1 hippocampus in kindled animals. The increase in this parameter meant the increment in occurrence of sEPSCs and glutamatergic transmission in CA1 hippocampus of kindled animals. These results supported previous reports which showed that NMDA receptor mediated responses in dentate gyrus granule cells (26,27) and CA3 pyramidal neurons (28) effectively increased for weeks after kindling (29). In addition, *in vitro* experiments revealed that mossy fibers sprouting correlated with an increase in recurrent excitation in the hippocampus of kindled animals (30,32). The observed increase in glutamatergic transmission in our study and previous mentioned reports might be related to the modification in AMPA and/or NMDA receptor functions as a result of variations in the open time of NMDA receptors and their block by Mg^2+^ (26), upregulation (33,34), and higher affinity of the NMDA and AMPA receptors (26,34,35) in epileptic neurons. 

The results of this study also revealed an opposite change in inter-event intervals of sIPSCs in fully kindled animals which meant a decrease in occurrence of GABA_A_ currents. In addition, the decay time constant of sIPSCs during returning to baseline decreased in the CA1 pyramidal cells of kindled animals. The decrease in time constant meant a faster return to baseline and faster decrease in GABAergic currents in kindled animals. Consistent with these results, it has been shown that GABAergic dependent inhibition decreases following temporal lobe epilepsy in CA1 and CA3 (36,40). In addition, GABA_A_ receptors in dentate gyrus are persistently altered following status epilepticus (41). The reduction of GABA receptors function and binding (39,42) and internalization of surface GABA receptors, perhaps via dephosphorylation of different subunits, may cause loss of inhibition in the CA1 area of the hippocampus after temporal lobe epilepsy (43,44). Therefore, the observed reduction in sIPSCs occurrence might be related to the above mechanisms. In addition, the reduction of GABA receptors binding (39,42) could be involved in the decreased decay-time constant of sIPSCs in kindled animals. 

The observed changes in sIPSCs might be affected by GABA_B_ receptors, A reduction in IPSCs mediated by GABA_B_ receptors following kindling has been reported (45,46). In addition, the efficacy of the presynaptic GABA_B_ receptors on GABAergic terminals of CA1 neurons decreased in hippocampal slices of kindled animals (40) which was consistent with downregulation of GABA_B_ presynaptic receptors (47). However, it must be noted that in our study QX-314 has been used in the internal solution during the sIPSC recording and this drug is a GABA_B_ receptor blocker. Therefore, the GABA_B_ receptors had no role in the recorded sIPSCs postsynaptically and its possible effects might be exerted through presynaptic mechanisms. 

In this study application of LFS prevented the kindling-induced changes in sEPSCs and sIPSCs in the kindled+LFS group. We previously showed that application of LFS has anticonvulsant effects in fully kindled rats (16). In addition, our previous experiments showed that application of LFS after each kindling stimulation (during epileptogenesis) prevented the seizure-induced changes in functional electrical properties of CA1 pyramidal neurons and decreased the excitability of these cells (18). However, the main difference between our present and previous study (18) was the time of the LFS application which occurred after the fully kindled state in the present study. The possible mechanisms of the LFS effects have been attributed to the accumulation of adenosine (48), secretion of brain-derived neurotrophic factor (49), elevation of cAMP (20), modification of receptor binding (50), and alteration of neuronal activity (51). In addition, it has been suggested that LFS exerts its inhibitory effects on kindled animals through mechanisms involved in long term depression (LTD) (52). Because the voltage dependent Ca^2+^ channels have an important role in LTD induction postsynaptically (53,54), and are the target of neuromodulation by adenosine (55), it is possible to consider a role for these channels in mediating the LFS effects on kindling-induced changes in synaptic transmission. However, further studies are required to reveal the exact mechanism(s) involved in the effect of LFS on sEPSCs and sIPSCs. 

## Conclusion

Taken together, the results of the present study showed that application of LFS in fully kindled animals could prevent the increase in spontaneous glutamatergic transmission and the decrease in spontaneous GABAergic transmission in hippocampal CA1pyramidal cells. This preventive effect of LFS could be considered as a mechanism for LFS anticonvulsive action in kindled animals. 
